# Roles for Exosome in Various Kidney Diseases and Disorders

**DOI:** 10.3389/fphar.2019.01655

**Published:** 2020-01-31

**Authors:** Visith Thongboonkerd

**Affiliations:** Medical Proteomics Unit, Office for Research and Development, Faculty of Medicine Siriraj Hospital, Mahidol University, Bangkok, Thailand

**Keywords:** acute kidney injury, chronic kidney disease, diabetic nephropathy, glomerular disease, lupus nephritis, nephrolithiasis, renal cell carcinoma, transplantation

## Abstract

Exosome is a nanoscale vesicle with a size range of 30–100 nm. It is secreted from cell to extracellular space by exocytosis after fusion of multivesicular body (MVB) (formed by endocytic vesicles) with plasma membrane. Exosome plays several important roles in cellular homeostasis and intercellular communications. During the last two decades, exosome has acquired a wide attention to explore its additional roles in various aspects of cell biology and function in several organ systems. For the kidney, several lines of evidence have demonstrated 1that exosome is involved in the renal physiology and pathogenic mechanisms of various kidney diseases/disorders. This article summarizes roles of the exosome as the potential source of biomarkers, pathogenic molecules, and therapeutic biologics that have been extensively investigated in many kidney diseases/disorders, including lupus nephritis (LN), other glomerular diseases, acute kidney injury (AKI), diabetic nephropathy (DN), as well as in the process of renal fibrosis and chronic kidney disease (CKD) progression, in addition to polycystic kidney disease (PKD), kidney transplantation, and renal cell carcinoma (RCC). Moreover, the most recent evidence has shown its emerging role in kidney stone disease (or nephrolithiasis), involving inflammasome activation and inflammatory cascade frequently found in kidney stone pathogenesis.

## Introduction

Exosome is a nanoscale extracellular vesicle with a size range of approximately 30–100 nm and spherical shape surrounded by lipid bilayers (Huotari and Helenius, 2011; Raimondo et al., 2011). Initially, exosome had been thought to serve only as an exocytic vesicle to shed some intracellular and membrane components out of the cells (Pan et al., 1985; Johnstone et al., 1987). However, recent evidence has revealed its more significant roles as the messenger cargo for intercellular communications (Hessvik and Llorente, 2018). Exosome is originated from small intraluminal vesicle inside multivesicular body (MVB) (also known as late endosome) that is subsequently fused with cellular plasma membrane to expel such nano-sized vesicle to the extracellular space (Hessvik and Llorente, 2018). Therefore, the exosome contains greater proportion of membrane proteins and MVB-formation proteins as compared to the parental cells (Bobrie et al., 2011; Colombo et al., 2014; Park et al., 2018). The biogenesis of exosome also enables transportation of various receptors, proteins, genetic materials [e.g., DNA, messenger RNA (mRNA), and microRNA (miRNA)] and lipids to the target cells, which incorporate these information-rich molecules from exosome by three major pathways, including receptor-ligand interaction, direct fusion with plasma membrane, and endocytosis (Bobrie et al., 2011; Colombo et al., 2014; Park et al., 2018).

Exosome can be found in several biological fluids, e.g., blood, seminal fluid, cerebrospinal fluid, synovial fluid, breast milk, saliva, bile, ascitic fluid, amniotic fluid, pleural fluid, and urine (Lin et al., 2015). For the renal system, recent studies have investigated the compositions of urinary exosomes secreted from different segments of the nephron and their relevance to the renal physiology and pathophysiology of kidney diseases (Morrison et al., 2016; Pomatto et al., 2017; Stahl et al., 2019). In addition, roles for exosomes derived from serum/plasma/blood, renal tubular cells, renal tissues, glomerular endothelial cells, mesenchymal stem cells (MSCs), urinary stem cells, cancer cells, and macrophages related to kidney diseases have been also examined. This review summarizes the current knowledge of roles for exosome in pathogenic mechanisms, biomarker discovery and therapeutics of various kidney diseases/disorders, particularly lupus nephritis (LN), other glomerular diseases, acute kidney injury (AKI), diabetic nephropathy (DN), as well as in the process of renal fibrosis and chronic kidney disease (CKD) progression, in addition to polycystic kidney disease (PKD), kidney transplantation, and renal cell carcinoma (RCC) (**Table 1**). Finally, the emerging role of exosome in kidney stone disease is also emphasized.

**Table 1:** Summary of roles for exosome in various kidney diseases/disorders.

Kidney disease/disorder	Role of exosome	Source of exosome	Main findings	Reference
LN	Biomarker	Urine (mouse)	Increased urinary exosomal miR-26a in LN animals with progressive glomerular injury.	(Ichii et al., 2014)
	Biomarker	Urine (human)	Increased miR-146a in urinary exosomes derived from SLE patients with active LN.	(Perez-Hernandez et al., 2015)
	Biomarker	Urine (human)	Urinary exosomal miR-29c might serve as a biomarker to predict renal fibrosis in LN.	(Sole et al., 2015)
	Biomarker	Urine (human)	Decreases of let-7 and miR-21 in urinary exosomes derived from LN patients.	(Tangtanatakul et al., 2019)
	Biomarker	Urine (human)	Urinary exosomal miR-3135b was increased in patients with active LN class IV compared to those with inactive LN class IV and healthy controls, whereas miR-654-5p was increased only in LN class IV with cellular crescent.	(Li et al., 2018b)
Other glomerular diseases	Biomarker	Urine (human)	Urinary exosomal aminopeptidase N, vasorin precursor, α-1-antitrypsin, and ceruloplasmin could differentiate IgA nephropathy from thin basement membrane nephropathy and healthy controls.	(Moon et al., 2011)
	Biomarker	Urine (human)	Urinary exosomal miR-193a was greater in children with primary focal segmental glomerulosclerosis comparing to those with minimal change NS.	(Huang et al., 2017)
	Biomarker	Urine (human)	Urinary exosomal excretion and *CCL2* mRNA level were increased in IgA nephropathy and correlated with the disease activity.	(Feng et al., 2018)
	Biomarker	Urine (human)	Thirty urinary exosomal miRNAs were markedly increased in children with idiopathic NS. Among these, miR-194-5p and miR-23b-3p correlated well with urine protein level.	(Chen et al., 2019)
	Biomarker	Urine (human)	Level of FSP1 in extracellular vesicles (including exosomes) was increased in patients with active crescentic glomerulonephritis and decreased after immunosuppressive therapy.	(Morikawa et al., 2019)
Ischemia/reperfusion-induced AKI	Pathogenic mechanism	Urine (rat)	Decreased urinary exosomal AQP-1 in animals with ischemia/reperfusion-induced AKI.	(Sonoda et al., 2009)
	Pathogenic mechanism	Urine (rat)	Decreased urinary exosomal AQP-1 and AQP-2 in animals with ischemia/reperfusion-induced AKI.	(Asvapromtada et al., 2018)
	Biomarker	Urine (rat)	- Increased urinary exosomal miR-16, miR-24, and miR-200c at an early (injury) phase of ischemia/reperfusion injury. - Increased urinary exosomal miR-125 and miR-351 at a late (fibrotic) phase of ischemia/reperfusion injury.	(Sonoda et al., 2019a)
	Therapeutics	MSCs (human)	Recovery of tubular damage in rats after administration of human Wharton’s jelly MSCs-derived extracellular vesicles.	(Zhang et al., 2014; Zhang et al., 2016)
	Therapeutics	MSCs (mouse)	High expression of exosomal CCR2 could reduce macrophage infiltration.	(Shen et al., 2016)
	Therapeutics	MSCs (rat)	Exosomes derived from adipose MSCs could protect ischemia/reperfusion-induced AKI.	(Lin et al., 2016)
	Therapeutics	Renal tubular epithelial cells (rat)	Intravenous administration of extracellular vesicles (mainly exosomes) derived from rat renal tubular cells could improve ischemia-induced renal injury.	(Dominguez et al., 2017)
	Therapeutics	Renal tubular epithelial cells (human)	Intravenous administration of exosomes derived from human renal tubular cells could improve ischemia-induced renal injury.	(Dominguez et al., 2018)
	Therapeutics	MSCs (human)	Exosomes derived from bone marrow MSCs were rich with miR-199a-3p and could prevent ischemia/reperfusion-induced AKI by increasing expression of miR-199a-3p in renal cells.	(Zhu et al., 2019a)
Sepsis-induced AKI	Biomarker	Urine (human)	Increased urinary exosomal NGAL and activating transcription factor 3 in sepsis-induced AKI patients.	(Panich et al., 2017)
	Biomarker	Renal tubular epithelial cells (mouse)	Increased exosomal miR-19b-3p in lipopolysaccharide-induced AKI.	(Lv et al., 2019)
	Therapeutics	Serum (mouse)/myoblast (mouse)	Exosomes derived from sera of animals with remote ischemic preconditioning and myoblasts under hypoxia and reoxygenation preconditioning could prevent sepsis-induced AKI *in vivo* and *in vitro*, respectively, by enhancing the expression of miR-21.	(Pan et al., 2019)
Cisplatin-induced AKI	Biomarker	Urine (rat)	Increase of fetuin-A but decrease of AQP-2 in urinary exosomes derived from cisplatin-induced AKI animals.	(Zhou et al., 2006; Sonoda et al., 2019b; Sonoda et al., 2019a)
	Therapeutics	MSCs (human)	Transfer IGF-1 receptor to cisplatin-treated renal tubular cells and co-incubation of these injured cells with soluble IGF-1 and exosomes derived from bone marrow MSCs significantly increased cell proliferation.	(Tomasoni et al., 2013)
	Therapeutics	MSCs (human)	Exosomes derived from umbilical cord MSCs could prevent cisplatin-induced AKI by 14-3-3ζ-dependent pathway.	(Jia et al., 2018)
DN	Pathogenic mechanism	Urine (human)	Increased urinary exosomal plasmin, prostasin and urokinase, and proteolytic activation of ENaC in type 1 DN patients.	(Andersen et al., 2015)
	Pathogenic mechanism	Glomerular endothelial cells (mouse)	Exosomes isolated from high-glucose-treated glomerular endothelial cells played a crosstalk role to activate EMT and fibrotic changes of mesangial cells and podocytes in DN.	(Wu et al., 2016; Wu et al., 2017)
	Pathogenic mechanism	Macrophages (mouse)	High-glucose enhanced secretion of exosomes from macrophages, which then caused mesangial proliferation and activated inflammatory cascade. Knockdown of TGF-β1 significantly reduced such effects of exosomes.	(Zhu et al., 2019b)
	Pathogenic mechanism/biomarker	Podocytes (mouse)/urine (human)	- AGEs activated TGF-β-Smad3 signaling pathway and induced secretion of Elf3-containing exosomes from the murine podocytes. - Urinary exosomal Elf3 was detectable only in DN patients, not in those with minimal change NS and healthy controls.	(Sakurai et al., 2019)
	Biomarker	Urine (human)	Urinary exosomal bikunin precursor and histone-lysine N-methyltransferase were increased, but voltage-dependent anion-selective channel protein 1was decreased in DN patients.	(Zubiri et al., 2014)
	Biomarker	Urine (human)	Urinary exosomal miR-320c, miR-6068, miR-1234-5p, miR-6133, miR-4270, miR-4739, miR-371b-5p, miR-638, miR-572, miR-1227-5p, miR-6126, miR-1915-5p, miR-4778-5p, and miR-2861 were increased, whereas miR-30d-5p and miR-30e-5p were decreased in type 2 DN patients.	(Delic et al., 2016)
	Biomarker	Urine (human)	Urinary exosomal miR-15b, miR-34a, and miR-636 were increased in type 2 DN patients.	(Eissa et al., 2016a)
	Biomarker	Urine (human)	Urinary exosomal miR-133b, miR-342, and miR-30a were increased in type 2 DN patients.	(Eissa et al., 2016b)
	Biomarker	Urine (rat)	Urinary exosomal miR-451-5p was increased in diabetic rats and could predict albuminuria at a later time-point.	(Mohan et al., 2016)
	Biomarker	Urine (human)	Urinary exosomal miR-150-5p, miR-362-3p, and miR-877-3p were increased, but miR-15a-5p was decreased in type 2 DN.	(Xie et al., 2017)
	Biomarker	Urine (human)	Increased urinary exosomal AQP-2 and AQP-5 in DN patients.	(Rossi et al., 2017)
	Biomarker	Urine (human)	Urinary exosomal *WT1* mRNA served as the diagnostic and prognostic marker for type 2 DN.	(Abe et al., 2018)
	Biomarker	Urine (human)	Urinary exosomal let-7c-5p was increased, whereas miR-29c-5p and miR-15b-5p were decreased in type 2 DN patients. These three miRNAs could predict the development of DN.	(Li et al., 2018a)
	Therapeutics	MSCs (rat)	MSCs-derived exosomes suppressed infiltration of dendritic cells into the kidney (by regulating expression of ICAM-1) and inhibited production of the pro-inflammatory cytokines (TNF-α and TGF-β1) and renal fibrosis.	(Nagaishi et al., 2016)
	Therapeutics	Urinary stem cells (human)	Exosomes derived from urinary stem cells carried growth factor, TGF-β1, angiogenin and bone morphogenetic protein-7, which might recover vascular regeneration and cell survival in an early phase of DN.	(Jiang et al., 2016)
Renal fibrosis and other CKD models	Pathogenic mechanism	Renal cortex (mouse)	TGF-β1 mRNA was transported by exosomes, which might activate fibroblast proliferation and development of renal fibrosis.	(Borges et al., 2013)
	Biomarker	Urine (human)	Urinary exosomal miR-29c was decreased and associated with degree of chronicity in CKD patients.	(Lv et al., 2013)
	Biomarker	Kidney (mouse)	Urinary exosomal miR-181a was decreased (200-fold) in CKD patients.	(Khurana et al., 2017)
	Biomarker	Kidney (mouse)	Increased level of secreting transglutaminase-2 (a fibrosis-activating enzyme) through exosomal pathway in UUO mice.	(Furini et al., 2018)
	Biomarker	Urine (human)	Urinary exosomal miR-29c was decreased and correlated with the degree of renal fibrosis.	(Chun-Yan et al., 2018)
	Biomarker	Urine (human)	Urinary exosomal miR-200b was decreased in CKD patients and the degree of decrease was greatest in urinary exosomes derived from cells other than those of proximal renal tubules.	(Yu et al., 2018)
	Biomarker	Urine (human)	Urinary exosomal miR-21 was increased in CKD patients and had a negative correlation with eGFR.	(Lange et al., 2019)
	Biomarker	Urine (human/rat)	Urinary exosomal ceruloplasmin was increased in CKD patients and animals with passive Heyman nephritis.	(Gudehithlu et al., 2019)
	Therapeutics	MSCs (human)	Exosome miR-let7c derived from MSCs could attenuate fibrotic process in renal tubular epithelial cells.	(Wang et al., 2016)
PKD	Pathogenic mechanism/biomarker	Urine (human)	Multiple gene products related to PKD were excreted into the urine *via* exosomal secretion.	(Pisitkun et al., 2004; Hogan et al., 2009)
	Biomarker	Urine (human)	Decreased levels of polycystin-1 and polycystin-2 but increased level of transmembrane protein 2 in urinary exosomes derived from ADPKD patients with *PKD1* mutation.	(Hogan et al., 2015)
	Biomarker	Urine (human)	Complement C3 and C9 were increased in urinary extracellular vesicles (including exosomes) derived from ADPKD patients with or without progressive CKD, whereas urinary exosomal villin-1, periplakin and envoplakin were increased only in ADPKD patients with progressive CKD.	(Salih et al., 2016)
	Biomarker	Urine (rat/human)	Increased urinary exosomal activator of G protein signaling 3 in PKD animals/patients.	(Keri et al., 2018)
	Biomarker	Urine (human)	Increased urinary exosomal CD133 in ADPKD patients.	(Bruschi et al., 2019)
Kidney transplantation	Pathogenic mechanism	Urine (human)	Increased levels of 50-kDa and 75-kDa subunits of γ-ENaC in urinary exosomes derived from albuminuric kidney transplant recipients.	(Hinrichs et al., 2018)
	Pathogenic mechanism	Urine (human)	Temporary decreased level of AQP**-**2 in urinary extracellular vesicles (including exosomes) on day 1 after kidney transplantation that might explain acute diuresis phenomenon.	(Oshikawa-Hori et al., 2019)
	Biomarker	Urine (human)	Increased urinary exosomal Na-K-2Cl co-transporter in cyclosporine-A-treated kidney transplant recipients.	(Esteva-Font et al., 2014)
	Biomarker	Urine (human)	Levels of Na^+^-Cl^−^ cotransporter and its phosphorylated form were greater in urinary exosomes derived from tacrolimus-treated kidney transplant recipients with hypertension.	(Rojas-Vega et al., 2015)
	Biomarker	Urine (human)	Higher level of CD3-positive urinary exosomes in patients with acute cellular rejection.	(Park et al., 2017)
	Biomarker	Urine (human)	Increased urinary exosomal tetraspanin-1 and hemopexin in patients with T-cell mediated rejection.	(Lim et al., 2018)
	Biomarker	Plasma (human)	Increased mRNA levels of *gp130, CCL4, TNFα, SH2D1B, CAV1,* and atypical chemokine receptor 1 in plasma exosomes derived from patients with antibody-mediated rejection.	(Zhang et al., 2017)
RCC	Pathogenic mechanism	Primary RCC cells (human)/RCC cell line (human)	RCC cells had increased exosomal TGF-β1 that further mediated natural killer cell dysfunction.	(Xia et al., 2017)
	Pathogenic mechanism/Biomarker	CSCs (human)	- CSCs-derived exosomes promoted proliferation and EMT of ccRCC. - CD103-positive exosomes served as the biomarker for metastatic ccRCC.	(Wang et al., 2019b)
	Biomarker	Urine (human)	Matrix metalloproteinase 9, ceruloplasmin, podocalyxin, Dickkopf-related protein 4 and carbonic anhydrase IX were increased, whereas AQP-1, extracellular matrix metalloproteinase inducer, neprilysin, dipeptidase-1, and syntenin-1 were decreased in urinary exosomes derived from RCC patients.	(Raimondo et al., 2013)
	Biomarker	Urine (human)	- Urinary exosomal miR-126-3p combined with miR-449a or miR-34b-5p could discriminate ccRCC from healthy individuals. - Urinary exosomal miR-126-3p combined with miR-486-5p could discriminate ccRCC from benign lesions.	(Butz et al., 2016)
	Biomarker	Urine (human)	Urinary exosomal miR-30c-5p served as the diagnostic marker for early-stage ccRCC.	(Song et al., 2019)
	Biomarker	Urine (mouse)	Urinary exosomal miR-204-5p served as the diagnostic marker for Xp11.2 translocation RCC.	(Kurahashi et al., 2019)
	Biomarker	Serum (human)	Serum exosomal miR-224 served as the prognostic marker for ccRCC.	(Fujii et al., 2017)
	Biomarker	Serum (human)	Serum exosomal miR-210 served as the diagnostic and prognostic marker for ccRCC.	(Wang X. et al., 2019)
	Biomarker	Serum (mouse)	Serum exosomal miR-126-3p, miR-17-5p and miR-21-3p were decreased 1-day after cryoablation of RCC.	(Zhang et al., 2018)
	Therapeutics	RCC cell line (human)	CD8+ T-cells stimulated by exosomes derived from RCC cells combined with GM-CSF and IL-12 exerted autologous anti-cancer effects.	(Xu et al., 2019)
Kidney stone disease	Pathogenic mechanism	Urine (human)	Stone formers excreted greater amount of urinary exosomes.	(Jayachandran et al., 2015)
	Pathogenic mechanism	Renal tubular epithelial cells (human)	Hyperoxaluria activated exosomal secretion from HK-2 cells.	(He et al., 2017)
	Pathogenic mechanism	Macrophages (human)	- Exosomes derived from COM-exposed macrophages had changes in levels of proteins involved in immune regulation, i.e., T-cell activation and homeostasis, Fcγ receptor-mediated phagocytosis, IFN-γ regulation, and cell migration. - Increased production of IL-1β (a marker for inflammasome activation) in exosomes derived from the COM-exposed macrophages that activated several functions of inflammatory cells, including monocytes, macrophages, and T-cells. - Knockdown of vimentin by siRNA could abolish the effects of exosomes derived from the COM-exposed macrophages on monocytic and T-cell migration and phagocytic activity of macrophages.	(Singhto et al., 2018)
	Pathogenic mechanism	Macrophages (human)	- Exosomes derived from COM-exposed macrophages had changes in levels of proteins involved in cytoskeletal and actin binding, calcium binding, stress response, transcription regulation, immune response, and ECM disassembly. - COM-induced exosomes caused IL-8 overproduction from renal tubular cells and enhanced neutrophil migration. - COM-induced exosomes could bind with COM crystals more tightly than the control exosomes and subsequently activated COM crystal invasion through the ECM.	(Singhto and Thongboonkerd, 2018)

## Exosome and the Renal Physiology

Kidney is one of the vital organs responsible for homeostasis to maintain and regulate the normal physiology of human body. Exosome has been proposed to play roles in cell-cell communications within nephron segments that affect the renal physiology (Stahl et al., 2019). Several proteomics studies have revealed that the majority of exosomes in the nephron are originated mostly from glomerular podocytes and tubular cells at proximal convoluted tubule, thick ascending limb of the Henle’s loop, distal convoluted tubule and collecting duct (Pisitkun et al., 2004; Miranda et al., 2010; Vlassov et al., 2012; Dear et al., 2013). In addition, there are numerous membrane vesicles with the diameter of 30–50 nm and positive MVB markers found in the pellet derived from the normal urine of healthy individuals (Pisitkun et al., 2004). Moreover, these urinary exosomes are enriched with proteins and transporters from each part of the nephron, e.g., podocin and podocalyxin from glomerular podocytes, aquaporin (AQP)-1 from proximal convoluted tubule, type-2 Na^+^-K^+^-2Cl^−^ cotransporter from thick ascending limb of the Henle’s loop, Na^+^-Cl^−^ cotransporter from distal convoluted tubule, and AQP-2 from collecting duct (Pisitkun et al., 2004).

The roles for exosome in the renal physiology have been investigated in both *in vitro* and *in vivo* settings. For water transport, exosomes secreted from vasopressin analogue-treated collecting duct cells expressed high level of AQP-2 and subsequently induced neighboring cells to up-regulate expression of AQP-2 to enhance water reabsorption (Street et al., 2011; Radin et al., 2012; Miyazawa et al., 2018). For intra-nephron communications, several studies have implicated that exosome is involved in proximal-distal signaling (Prunotto et al., 2013; Gildea et al., 2014; Merchant et al., 2017). For example, a proteomics study has demonstrated that exosomes derived from glomerular podocytes could pass through the upstream proximal renal tubule and transferred information to the downstream distal renal tubular epithelial cells (Prunotto et al., 2013). Similarly, another study has also shown that exosomes derived from proximal renal tubular cells treated with fenoldopam (a dopamine receptor agonist) could be transferred to distal renal tubule and collecting duct, and further modulated production of reactive oxygen species (ROS) in the cells lining these tubular segments (Gildea et al., 2014). In addition, exosomes derived from LLC-PK1 proximal renal tubular cells expressed glyceraldehyde-3-phosphate dehydrogenase (GAPDH), which could be delivered to distal convoluted tubule and collecting duct (Jella et al., 2016). GAPDH-expressing exosomes then induced conformational changes of epithelial sodium channel (ENaC) and reduced sodium ion reabsorption capability of the affected cells (Jella et al., 2016).

Apart from function involving the renal physiology, urinary exosomes also act as immune effectors to protect from bacterial infection in the urinary tract. The intact human urinary exosome carries several innate immune proteins, including lysozyme-C, dermcidin, mucin-1, calprotectin, and myeloperoxidase, all of which potently inhibit the growth of various pathogenic and non-pathogenic *Escherichia coli* strains (Hiemstra et al., 2014). Taken together, under the normal state of the kidney, urinary exosome acts as the shelter for bioactive molecules to effectively transmit the functional substances from the upstream renal tubular cells to the downstream tubular cells that finally influence functions or activities of the effector cells.

## Roles of Exosome in Various Kidney Diseases/Disorders

### Lupus Nephritis and Other Glomerular Diseases

To date, urinary exosome has been widely proposed as the promising source of non-invasive biomarkers for diagnostics and prognostics to predict severity and progression of various kidney diseases, including glomerular disorders (Hoorn et al., 2005). In a murine model of LN, it has been shown that miR-26a expression in urinary exosomes significantly increased along with progressive glomerular injury (Ichii et al., 2014). The data obtained from this study has implicated that miR-26a regulates podocyte differentiation and cytoskeletal integrity and its increase might serve as the marker for the injured podocytes in glomerulonephritis (Ichii et al., 2014). Transcriptomics study in humans has shown that miR-146a was increased in urinary exosomes derived from active LN patients and might serve as the diagnostic biomarker for discriminating active LN from healthy controls and systemic lupus erythematosus (SLE) patients without LN (Perez-Hernandez et al., 2015). In addition, early phase of renal fibrosis and chronicity in LN patients could be predicted by a decline in urinary exosomal miR-29c (Sole et al., 2015). Similarly, the anti-inflammatory miRNAs (i.e., let-7a and miR-21) were down-regulated in urinary exosomes derived from patients with LN and might serve as the non-invasive biomarkers to classify the clinical stage of LN (Tangtanatakul et al., 2019). Furthermore, urinary exosomal miR-3135b was increased in patients with active LN class IV as compared to those with inactive LN class IV and healthy controls, whereas miR-654-5p was increased only in LN class IV with cellular crescent (with very poor prognosis) (Li et al., 2018b).

For other glomerular diseases, a previous study using a proteomics approach has shown that urinary exosomal aminopeptidase N, vasorin precursor, α-1-antitrypsin, and ceruloplasmin could differentiate immunoglobulin A (IgA) nephropathy from thin basement membrane nephropathy and healthy controls (Moon et al., 2011). In another study, level of fibroblast-specific protein 1 (FSP1) has been shown to increase in extracellular vesicles (including exosomes) derived from patients with active crescentic glomerulonephritis (Morikawa et al., 2019). Interestingly, its level was decreased after immunosuppressive therapy (Morikawa et al., 2019).

In addition to proteins, exosomal mRNAs and miRNAs also serve as the valuable markers for glomerular disorders. For example, level of urinary exosomal miR-193a was greater in children with primary focal segmental glomerulosclerosis as compared to those with minimal change nephrotic syndrome (NS) (Huang et al., 2017). Urinary exosomal excretion and chemokine (C-C motif) ligand-2 (*CCL2*) mRNA level were increased in IgA nephropathy and correlated with the disease activity (Feng et al., 2018). Additionally, 30 urinary exosomal miRNAs were markedly increased in children with idiopathic NS (Chen et al., 2019). Among these, miR-194-5p and miR-23b-3p correlated well with urinary protein level (Chen et al., 2019). These data strengthen that urinary exosomes are rich with the information or molecules that can be further developed to serve as the diagnostic and prognostic biomarkers for LN and other glomerular diseases.

#### Acute Kidney Injury

Similar to LN, exosome has gained a wide attention as a source of pathogenic molecules, biomarkers, and therapeutic compounds in AKI. For pathogenic mechanisms and biomarker discovery, studies in a rat model have revealed that levels of urinary exosomal AQP-1 and AQP-2 protein and mRNA were reduced in animals with ischemia/reperfusion-induced AKI (Sonoda et al., 2009; Asvapromtada et al., 2018). The decreased levels of aquaporins in renal tubules could affect water handling of the nephron and might reflect the progressive development of AKI (Nielsen et al., 2002). In addition, different sets of exosomal miRNAs could be used as the biomarkers to classify AKI progression in ischemia/reperfusion injury. For example, the increased levels of miR-16, miR-24, and miR-200c were detected in urinary exosomes at an early (or injury) phase, whereas the increases of miR-125 and miR-351 were found at the late (or fibrotic) stage of ischemia/reperfusion injury (Sonoda et al., 2019a).

A most recent transcriptomics study has revealed that exosomal miR-19b-3p derived from renal tubular epithelial cells was elevated in mice with lipopolysaccharide-induced AKI (Lv et al., 2019). This molecule has been proposed to play a critical pathogenic role in tubulointerstitial inflammation by recruiting macrophage infiltration (Lv et al., 2019). Moreover, studies of cisplatin-induced AKI have revealed the increased level of fetuin-A but decreased level of AQP-2 in urinary exosomes from the AKI animals (Zhou et al., 2006; Sonoda et al., 2019a; Sonoda et al., 2019b). In another study, exosomes derived from patients with sepsis-induced AKI carried high amounts of neutrophil gelatinase-associated lipocalin (NGAL) and activating transcription factor 3 (Panich et al., 2017). All of these data indicate that changes in levels of the mentioned urinary exosomal proteins may serve as the biomarkers for diagnosis of AKI and prediction of its severity.

For therapeutics, there is increasing evidence implicating the renoprotective role of exosomes, particularly those derived from MSCs, for attenuation and/or prevention of AKI (Aghajani Nargesi et al., 2017). Administration of extracellular vesicles derived from human Wharton’s jelly MSCs could decrease renal injury and improve renal function after ischemia/reperfusion-induced renal damage in rats (Zhang et al., 2014; Zhang et al., 2016). High expression of C-C motif chemokine receptor-2 (CCR2) on exosomes derived from MSCs could reduce the level of its ligand (CCL2) and suppress its effects to induce macrophage infiltration in mice with ischemia/reperfusion-induced AKI (Shen et al., 2016). Exosomes derived from adipose MSCs could protect ischemia/reperfusion-induced AKI (Lin et al., 2016). Similarly, exosomes derived from bone marrow MSCs were rich with miR-199a-3p and could prevent ischemia/reperfusion-induced AKI by increasing expression of miR-199a-3p in renal cells (Zhu et al., 2019a). Additionally, intravenous administration of extracellular vesicles (mainly exosomes) derived from rat and human renal tubular cells could improve ischemia-induced renal injury (Dominguez et al., 2017; Dominguez et al., 2018).

In other models of AKI, exosomes derived from sera of animals with remote ischemic preconditioning and myoblasts under hypoxia and reoxygenation preconditioning could prevent sepsis-induced AKI *in vivo* and *in vitro*, respectively, by enhancing the expression of miR-21 (Pan et al., 2019). Also, transferring insulin growth factor-1 (IGF-1) receptor to cisplatin-treated renal tubular cells and co-incubation of these injured cells with soluble IGF-1 and exosomes derived from bone marrow MSCs significantly increased cell proliferation (Tomasoni et al., 2013). Moreover, exosomes derived from umbilical cord MSCs could prevent cisplatin-induced AKI *via* 14-3-3ζ-dependent pathway (Jia et al., 2018). Although all of these studies were carried out in animals, their results hold a promise for further validation in the clinical setting.

#### Chronic Kidney Disease

The roles of exosome have also been extensively investigated in CKD, particularly in DN and renal fibrosis models. A recent study has shown that type 1 DN was associated with increases in urinary excretion of exosomal plasmin, prostasin, and urokinase as well as the proteolytic activation of ENaC that might contribute to the dysfunction of Na^+^ excretion and hypertension (Andersen et al., 2015). Interestingly, exosomes derived from high-glucose-treated glomerular endothelial cells played a crosstalk role to activate epithelial mesenchymal transition (EMT) and fibrotic changes of glomerular mesangial cells and podocytes in DN (Wu et al., 2016; Wu et al., 2017). High-glucose also enhanced secretion of exosomes from macrophages, which then caused mesangial proliferation and activated inflammatory cascade in the renal tissue (Zhu et al., 2019b). Knockdown of TGF-β1 significantly reduced such effects of exosomes indicating that TGF-β1 serves as an important mediator for interactions or cross talks between macrophages and renal cells (Zhu et al., 2019b). In addition, advanced glycation end products (AGEs) activated TGF-β-Smad3 signaling pathway and induced secretion of a transcription factor Elf3 through exosomes from the murine podocytes (Sakurai et al., 2019). Moreover, urinary exosomal Elf3 was detectable only in DN patients, not in those with minimal change NS and healthy controls (Sakurai et al., 2019).

For biomarker discovery, urinary exosomal miR-451-5p was increased in diabetic rats at 6 weeks post-induction with streptozotocin and could predict albuminuria at a later time-point (Mohan et al., 2016). In addition, a proteomics study using label-free quantitative technique compared urinary exosomes derived from DN patients with those collected from healthy individuals. The data showed that urinary exosomal bikunin precursor and histone-lysine N-methyltransferase were increased, whereas voltage-dependent anion-selective channel protein 1 was decreased, in DN patients and might lead to the improve diagnostics and monitoring of DN (Zubiri et al., 2014). Later, the increased levels of AQP-2 and AQP-5 were detected in urinary exosomes derived from DN patients, suggesting that these proteins may serve as the non-invasive biomarkers for diagnosis of DN (Rossi et al., 2017). In a subsequent transcriptomics study using miRNAs microarrays, urinary exosomal miR-320c, miR-6068, miR-1234-5p, miR-6133, miR-4270, miR-4739, miR-371b-5p, miR-638, miR-572, miR-1227-5p, miR-6126, miR-1915-5p, miR-4778-5p, and miR-2861 were increased, whereas miR-30d-5p and miR-30e-5p were decreased in type 2 DN patients (Delic et al., 2016). Similarly, urinary exosomal miR-15b, miR-30a, miR-34a, miR-133b, miR-342, and miR-636 were increased in type 2 DN patients (Eissa et al., 2016a; Eissa et al., 2016b). *WT1* mRNA was increased in urinary exosomes derived from type 2 DN patients compared to those of patients with minimal change NS and healthy controls and might serve as the diagnostic and prognostic markers for type 2 DN (Abe et al., 2018). Contradictory changes in urinary exosomal miRNAs were also found. While miR-150-5p, miR-362-3p, and miR-877-3p were increased, miR-15a-5p was decreased in urinary exosomes derived from type 2 DN patients (Xie et al., 2017). Urinary exosomal let-7c-5p was increased, whereas miR-29c-5p and miR-15b-5p were decreased in type 2 DN patients, and all of these three miRNAs could predict the development of DN (Li et al., 2018a).

In renal fibrosis, a study using unilateral ureteric obstruction (UUO) model has indicated that transforming growth factor-β1 (TGF-β1) mRNA was transported by exosomes, which might activate fibroblast proliferation and development of renal fibrosis (Borges et al., 2013). Additionally, a recent proteomics study has demonstrated an increased level of secreting transglutaminase-2 (a fibrosis-activating enzyme) through exosomal pathway in UUO mice and suggested that its increase might serve as a prognostic biomarker to predict the progression of renal fibrosis (Furini et al., 2018).

In other studies of CKD, urinary exosomal miR-29c and miR-181a were decreased and correlated with the degree of chronicity or renal fibrosis in CKD patients (Lv et al., 2013; Khurana et al., 2017; Chun-Yan et al., 2018). Urinary exosomal miR-200b was decreased in CKD patients and the degree of decrease was greatest in urinary exosomes derived from cells other than those of proximal renal tubules (Yu et al., 2018). By contrast, urinary exosomal miR-21 was increased in CKD patients and inversely correlated with estimated glomerular filtration rate (eGFR) (Lange et al., 2019). Urinary exosomal ceruloplasmin was increased in animals with passive Heyman nephritis and also in CKD patients (Gudehithlu et al., 2019). Interestingly, its increase in animals was detected prior to the onset of proteinuria and thus might serve as an early biomarker to predict the renal histopathological change (Gudehithlu et al., 2019).

For therapeutics, the study in DN has shown that exosomes derived from bone marrow MSCs suppressed infiltration of dendritic cells into the kidney by regulating expression of intercellular adhesion molecule-1 (ICAM-1) (Nagaishi et al., 2016). These exosomes also inhibited renal fibrosis and reduced production of pro-inflammatory cytokines, i.e., tumor necrosis factor-α (TNF-α) and TGF-β1 (Nagaishi et al., 2016). In addition, exosomes derived from urinary stem cells carried growth factor, TGF-β1, angiogenin, and bone morphogenetic protein-7 that might play roles in vascular regeneration and cell survival in an early phase of DN (Jiang et al., 2016). In the UUO model, exosomal miR-let7c derived from MSCs could attenuate fibrotic process in TGF-β-treated renal tubular epithelial cells (Wang et al., 2016). Taken together, these findings indicate the potential roles of exosome for therapeutic intervention in CKD in both DN and UUO renal fibrosis models.

#### Polycystic Kidney Disease

In an early phase of proteomics studies of urinary exosomes, multiple gene products related to PKD were detected in the urine, implicating that they were excreted into the urine *via* exosomal secretion (Pisitkun et al., 2004; Hogan et al., 2009). Comparing to those derived from healthy individuals, urinary exosomes derived from autosomal dominant PKD (ADPKD) patients with *PKD1* gene mutation had decreased levels of polycystin-1 and polycystin-2, but increase of transmembrane protein 2 (Hogan et al., 2015). A subsequent proteomics study has demonstrated that complement components C3 and C9 were increased in urinary extracellular vesicles (including exosomes) derived from ADPKD patients with or without progressive CKD, whereas urinary exosomal villin-1, periplakin, and envoplakin were increased only in ADPKD patients with progressive CKD (Salih et al., 2016). More recently, the study in both animal model and in PKD patients has shown the increase in urinary exosomal activator of G protein signaling 3 (Keri et al., 2018). Furthermore, a most recent study has reported the increased urinary exosomal CD133 that could effectively discriminate ADPKD patients from those with medullary sponge kidney (Bruschi et al., 2019). These findings underscore the important role of exosomes as the source of the non-invasive biomarkers for diagnostics and prognostics of PKD.

#### Kidney Transplantation

Currently, renal biopsy is the gold standard for the diagnosis of kidney transplant rejection. However, such diagnostic procedure is invasive and is frequently accompanied by hematuria and other biopsy-related complications. Recently, much attention has been given to urinary and blood-derived exosomes as alternative means for monitoring the graft rejection. Indeed, exosomes served as the source of potential non-invasive biomarkers to facilitate the diagnosis of cell-mediated and antibody-mediated allograft rejection. The presence of intragraft infiltration of T-cells is one of the hallmarks for the diagnosis of acute cellular rejection after kidney transplantation. A recent study has shown the higher level of CD3-positive urinary exosomes in patients with acute cellular rejection that could reflect T-cell infiltration in the renal allografts (Park et al., 2017). Similarly, a subsequent proteomics study has demonstrated the increased levels of urinary exosomal tetraspanin-1 and hemopexin in patients with T-cell-mediated rejection (Lim et al., 2018). For antibody-mediated rejection, the increased mRNA levels of *gp130, CCL4, TNFα, SH2D1B, CAV1*, and atypical chemokine receptor 1 were found in plasma exosomes derived from patients with allograft rejection (Zhang et al., 2017).

Additionally, the information obtained from exosomes also leads to better understanding of the mechanisms and pathogenic events after kidney transplantation. For example, the increased levels of 50-kDa and 75-kDa subunits of γ-ENaC were found in urinary exosomes derived from albuminuric kidney transplant recipients and was associated with hypertension (Hinrichs et al., 2018). Moreover, the decreased level of AQP-2 was detected in urinary extracellular vesicles (including exosomes) 1-day after kidney transplantation together with increased urinary volume and decreased urinary osmolality (Oshikawa-Hori et al., 2019). However, such decrease of AQP-2 in urinary extracellular vesicles and diuresis were recovered by day-6 post-transplantation, suggesting the temporary decrease in the renal expression of AQP2 and impairment of renal tubular water handling during the first few days after transplantation that might explain the acute diuresis phenomenon after kidney transplantation (Oshikawa-Hori et al., 2019).

For biomarker discovery, exosomes could be used for monitoring toxicities from immunosuppressive drugs. For example, the increased urinary exosomal Na^+^-K^+^-2Cl^−^ cotransporter was observed in cyclosporine-A-treated kidney transplant recipients (Esteva-Font et al., 2014). Similarly, levels of Na^+^-Cl^−^ cotranspoter and its phosphorylated form were greater in urinary exosomes derived from tacrolimus-treated kidney transplant recipients with hypertension (Rojas-Vega et al., 2015). Interestingly, the increases in both of these cotransporters were associated with high blood pressure, which is a common complication of kidney transplantation during immunosuppressive therapy.

Overall, these findings strengthen the important roles of exosome as the source of pathogenic molecules and non-invasive biomarkers in kidney transplantation. Exosome-based monitoring during the early post-transplantation phase may help the clinicians to maintain and perhaps prolong the allograft survival and function.

#### Renal Cell Carcinoma

Exosome also plays roles in pathogenic mechanisms of RCC. For evasion of immune surveillance, an *in vitro* study has indicated that exosomes from primary RCC cells of patients with clear cell RCC (ccRCC) and from RCC cell line had increased level of TGF-β1 that further mediated natural killer cell dysfunction (Xia et al., 2017). For cancer progression, exosomes derived from cancer stem cells (CSCs) of RCC patients promoted proliferation and EMT of ccRCC (Wang et al., 2019b). Moreover, the same set of data also showed that CD103-positive exosomes served as the biomarker for metastatic ccRCC (Wang et al., 2019b).

In addition to its pathogenic role, exosome has been extensively investigated for biomarker discovery in RCC using body fluids, especially urine. From a previous proteomics study, matrix metalloproteinase 9, ceruloplasmin, podocalyxin, Dickkopf-related protein 4, and carbonic anhydrase IX were increased, whereas AQP-1, extracellular matrix metalloproteinase inducer, neprilysin, dipeptidase-1, and syntenin-1 were decreased in urinary exosomes derived from RCC patients (Raimondo et al., 2013). However, the majority of exosomal biomarker studies in RCC had focused on miRNAs. A study on urinary exosomes had shown that exosomal miR-126-3p combined with miR-449a or miR-34b-5p could discriminate ccRCC from healthy individuals (Butz et al., 2016). Moreover, urinary exosomal miR-126-3p combined with miR-486-5p could discriminate ccRCC from benign lesions (Butz et al., 2016). Similarly, other more recent studies have reported that urinary exosomal miR-30c-5p and miR-204-5p might serve as the diagnostic markers for early-stage ccRCC (in a human study) and Xp11.2 translocation RCC (in a murine model), respectively (Kurahashi et al., 2019; Song et al., 2019). In addition, two consistent studies in humans have revealed that serum exosomal miR-224 and miR-210 could serve as the prognostic and diagnostic biomarkers for ccRCC (Fujii et al., 2017; Wang X. et al., 2019). Furthermore, serum exosomal miR-126-3p, miR-17-5p and miR-21-3p were decreased 1-day after cryoablation of RCC in a murine model and might be used as the biomarker for monitoring therapeutic outcome in RCC (Zhang et al., 2018).

For therapeutics, the *in vitro* study on a RCC cell line showed that CD8^+^ T-cells stimulated by exosomes derived from RCC cells combined with granulocyte-macrophage colony-stimulating factor (GM-CSF) and interleukin (IL)-12 exerted autologous anti-cancer effects (Xu et al., 2019). Taken together, exosomes play several roles in RCC, from the pathogenesis to diagnostics, prognostics and therapeutics.

#### Emerging Role of Exosome in Kidney Stone Disease

Kidney stone disease (“nephrolithiasis” or “urolithiasis”) is a common but under-estimated disease of the world involving 1–20% of worldwide population, depending on geographical areas (Thongboonkerd, 2008; Vinaiphat and Thongboonkerd, 2017). Its incidence/prevalence has been increasing in both industrialized and developing countries (Ziemba and Matlaga, 2017; Alelign and Petros, 2018). Calcium oxalate (CaOx) is the most common constituent in kidney stone matrix accounting approximately 80% of all kidney stones analyzed (Schubert, 2006). The stone formation frequently starts with supersaturation of urinary calcium and oxalate ions to form CaOx crystals inside the renal tubules (Thongboonkerd et al., 2006). After crystallization, CaOx crystalline particles can increase their sizes by crystal growth and self-aggregation and then be retained inside renal tubules by crystal-cell adhesion (Tsujihata, 2008).

Between the two common hydrated forms of CaOx crystals, CaOx monohydrate (COM) is more prominent and more pathogenic than CaOx dihydrate (COD) in kidney stone disease (Semangoen et al., 2008a; Semangoen et al., 2008b; Thongboonkerd et al., 2008; Vinaiphat et al., 2017; Peerapen et al., 2018). By differences in adhesive capability, atomic lattice, binding kinetics and surface atomic pattern of these two hydrated forms, COM crystal adheres more tightly to renal epithelial cell surface and is then internalized into the cells and subsequently transferred to the renal interstitium (Chiangjong and Thongboonkerd, 2012; Kanlaya et al., 2013; Chaiyarit et al., 2016; Chiangjong and Thongboonkerd, 2016; Vinaiphat et al., 2017). In addition to forming stone(s) in the renal interstitial locale (Randall’s plaque’s model) (Alelign and Petros, 2018; Wiener et al., 2018), the crystals deposited in the renal interstitium can then trigger several cascades of cellular response, e.g., increases in production of prostaglandin E, ROS, and inflammatory chemokines/cytokines (Umekawa et al., 2002; Khan, 2004; Khan, 2014; Mulay et al., 2014). In particular, the increased synthesis of chemokines [i.e., osteopontin, MIP-1 (macrophage inflammatory protein-1), RANTES (regulated upon activation normal T cell expressed and secreted), and MCP-1 (monocyte chemoattractant protein-1)] in the renal interstitium can activate and attract monocytes, macrophages, and other leukocytes to the crystal-deposited locales to serve as effector cells involving the inflammatory processes (Umekawa et al., 2002; Okada et al., 2010).

Although many efforts have been made to extensively investigate the roles for exosome in several other kidney diseases, the contributions of exosome in kidney stone disease remain under-investigated (**Table 1**). Indeed, the involvement of exosome-like vesicles in kidney stone disease had initially been discovered in rat models (Nagasawa et al., 1992; Khan et al., 2012). These studies have suggested that crystal deposition in renal papillae might start from nanoscale membrane vesicles derived from brush border of the injured renal cells that could induce nucleation, crystallization, and growth of the causative crystals at the periphery within a collagen framework (Khan et al., 2012). In humans, patients with kidney stones (stone formers) showed differential levels of urinary exosomal secretion when compared with the healthy volunteers without kidney stone (Jayachandran et al., 2015). Later, additional evidence has suggested that hyperoxaluria (an aggravating or etiologic factor for kidney stone formation) caused cytotoxicity in HK-2 cells and activated exosomal secretion (He et al., 2017). Nevertheless, the biological relevance of the increased exosomal secretion in kidney stone disease remains under-investigated.

Macrophage is an effector cell responsible for elimination of CaOx crystals deposited in the renal interstitium but, on the other hand, can aggravate or worsen tissue inflammation in kidney stone disease by autocrine, paracrine, and/or cytokine mechanisms (Kusmartsev et al., 2016). Recently, a combined expression and functional study has been conducted to examine proteome changes in exosomes derived from macrophages after exposure to COM crystals using a gel-based proteomics technology followed by several functional assays (Singhto et al., 2018). The data have shown that exosomes derived from COM-exposed macrophages had changes in levels of proteins involved in immune regulation, i.e., T-cell activation and homeostasis, Fcγ receptor-mediated phagocytosis, interferon-γ (IFN-γ) regulation, and cell migration (Singhto et al., 2018). Functional assays revealed an increase in production of IL-1β (a marker for inflammasome activation) in exosomes derived from the COM-exposed macrophages. Additionally, these exosomes activated several functions of inflammatory cells, including monocytes, macrophages, and T-cells. Moreover, exosomes derived from the COM-exposed macrophages caused an increase in production of pro-inflammatory cytokine (IL-8) in monocytes. Interestingly, knockdown of expression of vimentin (one of the significantly increased proteins in exosomes derived from the COM-exposed macrophages identified by proteomics approach) by small-interfering RNA (siRNA) could abolish the effects of exosomes derived from the COM-exposed macrophages on monocytic and T-cell migration and phagocytic activity of macrophages (Singhto et al., 2018). The data obtained from this study indicate that macrophage-derived exosome is involved, at least in part, in immune process and inflammatory cascade frequently found in kidney stone pathogenesis.

In addition to the inflammasome activation, a most recent study using label-free, gel-free, quantitative proteomics approach has identified 26 proteins whose levels were significantly changed in exosomes derived from the COM-exposed macrophages as compared to the control exosomes derived from the untreated macrophages (Singhto and Thongboonkerd, 2018). These proteins with significantly altered levels were involved mainly in cytoskeletal and actin binding, calcium binding, stress response, transcription regulation, immune response, and extracellular matrix (ECM) disassembly. Functional assays have shown the IL-8 overproduction from renal tubular cells treated with these COM-induced exosomes that also enhanced neutrophil migration. In concordance, these COM-induced exosomes were more fragile, thereby were easily to release their intraluminal contents for inflammasome activation. Interestingly, these COM-induced exosomes could bind with COM crystals more tightly than the control exosomes and subsequently activated COM crystal invasion through the ECM (Singhto and Thongboonkerd, 2018), a process that relies on plasminogen-plasmin activity on the COM crystal surface (Chiangjong and Thongboonkerd, 2012; Chiangjong and Thongboonkerd, 2016). These findings strengthen the pathogenic roles of macrophage-derived exosome, particularly in the inflammatory cascade of kidney stone disease induced by COM crystals in the renal interstitium.

## Conclusions and Future Perspectives

The aforementioned studies have demonstrated the important roles for exosome as the vesicular cargo for carrying and transferring molecular mediators for cell-cell communications and signal transduction. For example, exosomes derived from the urinary system are involved in intercellular communications under the physiologic and pathologic states. Their release during development and progression of kidney diseases may exert dual effects, i.e., aggravating the disease severity while promoting tissue repair. Exosome not only plays a role in the pathogenic mechanisms of kidney diseases but also serves as the valuable source of potential non-invasive biomarkers for diagnostics and prognostics (Hoorn et al., 2005). Comparing to the conventional urinary and circulating biomarkers, exosome carries and is enriched with specific sets of biomarker molecules, particularly receptors, proteins, genetic materials (e.g., DNA, mRNA, and miRNA) and lipids that are much less abundant in the urine and circulating blood. Therefore, exosomal markers provide the advantages for biomarker discovery in specific diseases that involve abnormalities of such molecules carried by exosome. Because these biomarker molecules are carried inside the exosomal cargo, they are more stable in biological fluids as compared to the other freely circulating molecules. In addition to serving as the important source of non-invasive biomarkers, exosome also provides the therapeutic potential as well (Tomasoni et al., 2013; Nagaishi et al., 2016; Aghajani Nargesi et al., 2017). Details of the roles for exosomes in various kidney diseases/disorders obtained from previous and recent studies are summarized in **Table 1**. Nevertheless, it should be noted that exosomes have not yet entered into the clinical practice, which of course needs clinical trials and validation in large cohorts.

In kidney stone disease, the emerging role of exosome has been recently reported to serve as the mediator to promote kidney stone generation at the initial phase of inflammatory cascade by activation of inflammasome (Singhto and Thongboonkerd, 2018; Singhto et al., 2018). Although the mentioned recent studies have provided convincing evidence for the pathogenic role of macrophage-derived exosome in kidney stone disease, there are still many challenges that need further elucidations. For example, roles of exosomes derived from cells other than macrophages (e.g., renal cells aligning various segments of the nephron) should be also investigated. In addition, large-scale analyses of exosomes in kidney stone research should not be limited only to proteomics but can be done by other “omics” studies (e.g., transcriptomics, lipidomics, metabolomics, interactomics, etc.) as well. Moreover, it would be interesting to observe the effects of exosomes derived from specific cell types on the disease course using animal models or *ex vivo* setting. However, isolation or purification of exosomes from animal model or raw biological fluids is still challenging because some components, such as high-density lipoproteins, chylomicrons, and microvesicles, have their size ranges close to that of exosomes (Yuana et al., 2014). Recently, several efforts have been made to optimize the isolation/purification of exosomes (Gheinani et al., 2018; Ayala-Mar et al., 2019; Doyle and Wang, 2019; Hou et al., 2019; Huang et al., 2019; Wang et al., 2019a; Yang et al., 2019). Nevertheless, the outcome is still unsatisfactory. Therefore, it is essential to further optimize the isolation methods or develop new techniques to isolate/purify exosomes more efficiently. Finally, genetic manipulation of specific transcript or protein compositions of the exosome would yield better understanding of the mechanisms for exosomal involvement in kidney stone disease. Having done so, the roles for exosome in kidney stone disease will be much clearer.

## Author Contributions

The author confirms being the sole contributor of this work and approved it for publication.

## Conflict of Interest

The author declares that the research was conducted in the absence of commercial or financial relationships that could be construed as a potential conflict of interest.

## Abbreviations

ADPKD, autosomal dominant polycystic kidney disease; AGEs, advanced glycation end products; AKI, acute kidney injury; AQP, aquaporin; CaOx, calcium oxalate; CCL2, chemokine (C-C motif) ligand-2; CCR2, C-C motif chemokine receptor-2; ccRCC, clear cell renal cell carcinoma; CKD, chronic kidney disease; COD, calcium oxalate dihydrate; COM, calcium oxalate monohydrate; CSCs, cancer stem cells; DN, diabetic nephropathy; ECM, extracellular matrix; eGFR, estimated glomerular filtration rate; EMT, epithelial mesenchymal transition; ENaC, epithelial sodium channel; FSP1, fibroblast-specific protein 1; GAPDH, glyceraldehyde-3-phosphate dehydrogenase; GM-CSF, granulocyte-macrophage colony-stimulating factor; ICAM-1, intercellular adhesion molecule-1; IGF-1, insulin growth factor-1; IFN-γ, interferon-γ; IL, interleukin; LN, lupus nephritis; MCP-1, monocyte chemoattractant protein-1; MIP-1, macrophage inflammatory protein-1; miRNA, microRNA; MSCs, mesenchymal stem cells; MVB, multivesicular body; NGAL, neutrophil gelatinase-associated lipocalin; NS, nephrotic syndrome; PKD, polycystic kidney disease; RANTES, regulated upon activation normal T cell expressed and secreted; RCC, renal cell carcinoma; ROS, reactive oxygen species; siRNA, small-interfering RNA; SLE, systemic lupus erythematosus; TGF-β1, transforming growth factor-β1; TNF-α, tumor necrosis factor-α; UUO, unilateral ureteric obstruction.
